# Therapeutic strategies and nano-drug delivery applications in management of ageing Alzheimer’s disease

**DOI:** 10.1080/10717544.2018.1428243

**Published:** 2018-01-19

**Authors:** Govindarajan Karthivashan, Palanivel Ganesan, Shin-Young Park, Joon-Soo Kim, Dong-Kug Choi

**Affiliations:** a Department of Biotechnology, College of Biomedical and Health Science, Research Institute of Inflammatory Diseases Konkuk University, Chungju, Republic of Korea;; b Department of Applied Life Science, Graduate school of Konkuk University, Chungju, Republic of Korea;; c Nanotechnology research center, College of Biomedical and Health Science, Konkuk University, Chungju, Republic of Korea

**Keywords:** Alzheimer’s, nanodrugs, molecular targets, acetylcholine, oxidative stress, CNS

## Abstract

In recent years, the incidental rate of neurodegenerative disorders has increased proportionately with the aging population. Alzheimer’s disease (AD) is one of the most commonly reported neurodegenerative disorders, and it is estimated to increase by roughly 30% among the aged population. In spite of screening numerous drug candidates against various molecular targets of AD, only a few candidates – such as acetylcholinesterase inhibitors are currently utilized as an effective clinical therapy. However, targeted drug delivery of these drugs to the central nervous system (CNS) exhibits several limitations including meager solubility, low bioavailability, and reduced efficiency due to the impediments of the blood-brain barrier (BBB). Current advances in nanotechnology present opportunities to overcome such limitations in delivering active drug candidates. Nanodrug delivery systems are promising in targeting several therapeutic moieties by easing the penetration of drug molecules across the CNS and improving their bioavailability. Recently, a wide range of nano-carriers, such as polymers, emulsions, lipo-carriers, solid lipid carriers, carbon nanotubes, metal based carriers etc., have been adapted to develop successful therapeutics with sustained release and improved efficacy. Here, we discuss few recently updated nano-drug delivery applications that have been adapted in the field of AD therapeutics, and future prospects on potential molecular targets for nano-drug delivery systems.

## 1. Introduction

Alzheimer’s Disease (AD) is a progressive neurodegenerative disorder characterized by memory, cognition and behavioral impairment (dementia), and it eventually leads to mood fluctuation and fatal delirium (Fong et al., 2009). The increasing proportion of ageing inhabitants has escalated AD as the most predominant neurodegenerative disorder. AD is a worldwide concern, and nearly 36 million inhabitants were reported with dementia-AD since 2010, which was projected to double by 2030 with around 65.7 million clinical cases (Wortmann, 2013). Numerous clinical data suggested that AD patients exhibit severe impairment of cholinergic–neurotransmitter systems, possibly due to the suppression of acetylcholine (responsible for neural synapse) by Acetylcholinesterase (AChE) activity (Birks, 2006; Grothe et al., 2013). Subsequently, accumulating evidence also suggested that the activation of the glutamatergic system plays a significant role in AD pathology (Danysz & Parsons, 2012; Revett et al., 2013). Based on these two scientific leads, researchers have developed few approved drug candidates such as tacrine, donepezil, rivastigmine, galantamine (AChE inhibitors), and memantine (NMDA inhibitor) (Qizilbash et al., 2000; Ferris & Farlow, 2013; Birks et al., 2015). However, the pharmacokinetic (half-life) profiles of the drugs in biological system have been found to be potentially low and have also exhibited substantial side-effects (Wagstaff & McTavish, 1994; Kavirajan & Schneider, 2007; Hansen et al., 2008). Despite that, numerous pharmaceutical bodies such as Merck & Co., Lily, Pfizer, etc., have invested millions of dollars in AD therapeutic-clinical trials in recent years. However, there have been setbacks due to a withdrawal/hold of their top most effective drug candidates during phase II/III human clinical trials. The reason reported for this withdrawal was “virtually no chance of finding a positive clinical effect” (Mullard, 2017). Though these drug candidates were successful in pre-clinical studies, they failed to exhibit the anticipated efficacy in human trials. The potential reasons behind these therapeutic failures can be due to poor pharmacokinetics or low bioavailability, chemical nature (absorption in biological – blood brain barrier system), volatility (oxidation, hydrolysis) of evaluated drug candidates in biological system (Becker et al., 2008; Mullard, 2017; Sharma & Singh, 2011).

Applying nanotechnology in drug delivery systems has improve the bioavailability and kinetic profile of drugs in biological systems (Parveen & Sahoo, 2006). Advances in nanotechnology aid in targeting drugs to specific molecular targets and safely delivering drugs to specific sites of action (Roney et al., 2005; Blasi et al., 2007). The sustained release of nano-drug delivery systems enhances the controlled release profile of loaded drugs, thereby minimizing the dosage-regimen (Kumari et al., 2010). Thus, the overall effectiveness of drug candidates can be enhanced by adapting nano-drug delivery systems in AD therapeutics. In this review, we discuss recent updates and findings on the various nano-drug delivery systems adopted in the field of AD therapeutics, and future prospects of potential targets for AD nano-therapies.

## 2. Nanoparticles in drug delivery system and its importance

Nanoparticles (NP) are colloidal elements incorporated with active drug candidates, and these are considered to be a single entity that expresses controlled release/site-specific drug delivery in biological systems (Singh & Lillard, 2009). Conventional drug delivery including powder, capsule, tablet or liquid have crucial limitations including a high-dose requirement, low bioavailability, rapid first pass metabolism, and poor-pharmacokinetics (Mudshinge et al., 2011). Indeed, some bioactive candidates, such as polyphenols, proteins, and peptides, were documented to be poorly soluble and less absorbed in gastrointestinal systems, and this accounts for their weak therapeutic efficacy in biological systems and associated failure in clinical trials (Munin & Edwards-Lévy, 2011; Hajieva, 2017). NPs overcome these hurdles by safely transporting the drug across the acidic biological milieu with improved permeability, thereby delivering maximum efficacy at a relatively lower dose^94^. In addition, due to their malleable physicochemical properties, even a poorly soluble drug candidate can also be effectively translocated to the targeted site with an improved pharmacokinetic/bioavailability profile (Singh & Lillard, 2009; Mudshinge et al., 2011).

Substantial research has reported the proactive role of NPs to bypass oral and intestinal absorption barriers and deliver drugs to the site of action (Ensign et al., 2012; Lundquist & Artursson, 2016). This has been achieved due to its smaller size (typically ranging from one to several hundred nanometers), appropriate charge with a higher surface-volume ratio (Singh & Lillard, 2009; Zaman et al., 2014). In the case of the neuronal system, an additional layer of protection, the blood brain barrier, still persists as a major hurdle for various nanodrugs targeting neuronal systems (Misra et al., 2003; Pavan et al., 2008). Altering the characteristics associated with surface modulation favors NPs to cross through the blood brain barrier (BBB) by avoiding phagocytic opsonization, thereby improving the drug concentration in the brain (Gidwani & Singh, 2014; Zaman et al., 2014). For instance, particulates that are lipophilic with lower molecular weight (less than 400 Da) and size below 100 nm can pass through the BBB through diffusion mechanisms (Pajouhesh & Lenz, 2005). The micrometer scale of average human cells and blood capillaries indicates the importance of nano-particulate transport in biological systems (Pajouhesh & Lenz, 2005; Pardridge, 2005).

## 3. Nano-drug delivery systems in AD therapeutics

Nanotechnology, in association with therapeutics, helps to overcome several hurdles faced by drug candidates in treating neurodegenerative disorders, i.e. AD, PD (Grossman et al., 2009; Serafini, 2017). For instance, in AD therapeutics, orally administered drug moieties are anticipated to pass through oral-cum-gastric pH barriers, effective absorption, sustainable circulation in blood stream accompanied with successful transport across the blood brain barrier (BBB), reaching the targeted site of action (Alavijeh et al., 2005; Stegemann et al., 2007). Early breakdown of drug molecules in the alimentary system leads to a quick elimination of the drug, with a reduced half-life and poor bioavailability to obtain the anticipated therapeutic effects. In addition, prolonged interaction or inadvertent activation of drug molecules at unspecific targeted sites leads to prevalence of various adverse effects, such as abdominal complications, vomiting, nausea etc. (Kratz, 2008; Sainsbury et al., 2014). The physico-chemical traits of the drug candidates, including the molecular weight, net-charge, polarity, solubility, affinity for hydro or lipids moieties, have been documented to play a vital role in therapeutic failure of drugs (Alyautdin et al., 2014; Sainsbury et al., 2014). Nanotechnology in drug delivery system drastically modulates these properties of the drug candidates and improves the therapeutic potential using various functional carrier systems (Re et al., 2012; Spuch et al., 2012).

**Figure 1.**
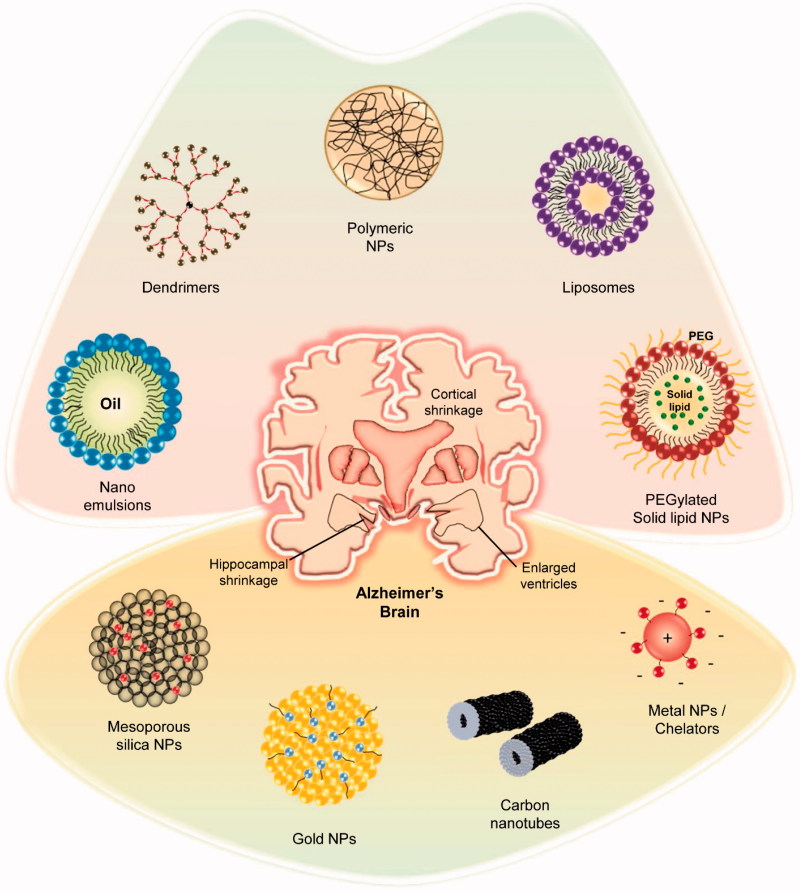
Graphical representation of few successful nano-carriers adapted in AD nano-therapeutics.

Based on the nature of the carrier material, nano-delivery systems can be roughly classified as organic (liposomes, polymers, emulsions, solid-lipid NPs, and dendrimers) and inorganic (silica, carbon, and gold), as depicted in Figure 1. Herein, we discuss nano-drug delivery applications for AD therapeutics. A list of few nano-drugs that were successfully developed and the associated nanocarriers is shown in Table 1.

**Table 1.** A list of few potential nano-drug delivery systems investigated in the field of AD therapeutics.

Nanodrug-delivery systems	Carrier material	Active drug candidate	Investigation model	Molecular targets	Comments
Carbon nanotubes (CNTs)	Pristine Multi-walled (MW)CNTs; Phospholipids and polysorbates	Berberine	β-amyloid induced AD in wistar rats	BBB transcytosis, Cholinergic systems	BRB-loaded MWCNT coated with phospholipids and polysorbates, significantly restored the memory impairment and suppressed ACHE activity in AD rats, compare to its free form. (Lohan et al., 2017)
Dendrimers	Hydrophobic pyridylphenylene dendrimers	*o*-phenylene diamine	Inclusion bodies of ovine prion protein (PrP) representing amyloid protein aggregates	Amyloid cascades	The dendrimers form stable protein complexes, thereby resultantly disrupt the PrP amyloid aggregates at a physiological pH of 7.4, which can be adapted in reducing Aβ burden. However further animal studies are required. (Sorokina et al., 2016)
Gold (Au) NPs	AuNPs of 5 nm with PEG	Anthocyanin	Mouse brain endothelial cells/Aβ1–42 Mouse Model	Amyloid cascades and tau hyperphosphorylation	Anthocyanin-loaded PEG-AuNPs effectively exhibited neuroprotective potential compared to its free form, via regulation of p-PI3K/p-Akt/p-GSK3β pathway, inhibition of tau hyperphosphorylation and amyloid cascades in AD mice model. (Ali et al., 2017)
Gold (Au) NPs	Gold colloids – rods (AuNR) and spheres (AuNS)	CLPFFD peptide	Porcine brain capillary endothelial cells	Amyloid cascades	PEGlyation of Au NPS, effectively stabilize the NPs by masking its negative charge and facilitates BBB transport. Further functionalizing with CLPFFD peptide enhances their selective binding towards Aβ-amyloid fibrils. (Ruff et al., 2017)
Liposome NPs	Sphingomyelin, cholesterol – functionalized with dimyristoylphosphatidic acid (PA) or bifunctionalized with PA and modified Apolipoprotein E-derived peptide (mApo)	Curcumin derivative (CD)	Human blood plasma and cerebrospinal fluid (*ex vivo*)	Amyloid cascades	mApo-PA-functionalized NPs effectively bind with Aβ1–42 in human biological fluid, which shall be adapted in promoting ‘sink effect’ in reducing Aβ burden. (Conti et al., 2017)
Liposome NPs	PLGA [Poly (lactic-co-glycolic acid)] – functionalized with anti-transferrin receptor monoclonal antibody (OX26) and anti-Aβ	Peptide iAβ5	Porcine brain capillary endothelial cells	BBB transcytosis, Amyloid cascades	Functionalized PLGA NPs safely and efficaciously transport iAβ5 peptides across BBB cell models and can be targeted to Aβ burden. (Loureiro et al., 2016)
Liposome NPs	Cholesterol, soybean phosphatidylcholine-functionalized with surface wheat germ agglutinin (WGA) and Cardiolipin (CL)	Curcumin and nerve growth factor	Human neuroblastoma cell Line/AD rat model	BBB transcytosis, Amyloid cascades and tau hyperphosphorylation	Curcumin-CL NPs inhibited phosphorylation of p38, JNK, and tau protein in Aβ insulted neurons. WGA-curcumin-CL NPs substantially reduced Aβ plaque deposition and lowered AChE activity in the hippocampus of AD rats. (Kuo et al., 2017)
Mesoporous silica NPs (MSN)	*N*-Cetyltrimethyl ammonium bromide – functionalized with succinic anhydride (S) and 3-aminopropyltriethoxysilane (A)	Rivastigmine hydrogen tartrate (RT)	Simulated gastric and body fluids and neuroblastoma SH-SY5Y cell line viability	Neuronal cell death/Cholinergic systems	RT-A-MSNs exhibited a sustained release profile of RT in gastric and body fluids. However, at higher dose concentrations, the bioaccumulation of RT-A-MSNs may be higher which resultantly shows toxicity to neuronal cells, compared to other functionalized MSNs. Thus, further extensive *in vivo* studies are required. (Karimzadeh et al., 2017)
Mesoporous silica NPs (MSN)	*N*-Cetyltrimethylammonium bromide, Tetraethoxysilane – functionalized with gold (Au) nanoparticle	Metal chelator CQ (5-chloro-4-hydroxy-7-iodoquinoline)	PC12 rat adrenal medulla cells/endothelial cell line.	BBB transcytosis, Amyloid cascades	MSN-AuNPs were reported to effectively cross *in vitro* model of the BBB and the metal chelator CQ loaded MSN-AuNPs significantly inhibits Cu^2+^-induced Aβ40 aggregation. (Yang et al., 2016)
Metallic NPs	Iron crystal structure; functionalized with PEG	Iron oxide	Amyloid fibrillation experiments – *in vitro*	Amyloid cascades	Under magnetic field, the higher concentration of NPs accelerates Aβ fibrillation, whereby at lower concentration inhibits the same. Interestingly negative charged or uncharged NPs inhibits fibrillation more efficiently. (Mirsadeghi et al., 2016)
Polymeric NPs	Dendrigraft poly-l-lysines and polyethelene glycol (PEG)	RVG29 peptide and BACE1-AS shRNA gene	Double transgenic AD mice/neuroblastoma and brain capillary endothelial cell line	Amyloid cascades and tau tangles	These multifunctional nanocarriers, successfully delivered dual therapeutic drug moieties and effectively suppressed Aβ plaque burden and obstruct phosphorylated-tau-tangles formation. (Liu et al., 2016)
Polymeric NPs	Chitosan	Piperine	Colchicine induced AD rats	Cholinergic and oxidative stress systems	Compare to its free form, piperine NPs effectively alleviated the behavioral impairment in AD rat model via suppressing AChE and oxidative stress environment. (Elnaggar et al., 2015)
Polymeric NPs	Glycidyl methacrylate	Iminodiacetic acid (IDA)	Zinc-mediated Aβ42 aggregation/human neuroblastoma SH-SY5Y cell line viability	Amyloid cascades	IDA-NPs effectively chelated zinc mediated Aβ42 aggregates, thereby proposed to strongly inhibit Aβ42 fibrillation pathway. However further animal studies are required to confirm the same. (Liu et al., 2017a)
Polymeric NPs	*N*-isopropyl acrylamide and *N*-t-butyl acrylamide – monomers; acrylic acid	Epigallocatechin-3-gallate (EGCG)	Amyloid fibrillation experiments – *in vitro* and neuroblastoma SH-SY5Y cell line viability	Amyloid cascades	Negatively charged polymeric NPs loaded with EGCG synergistically suppressed Aβ (Aβ42 and Aβ40) fibrillation. However, further animal studies are required to confirm the same. (Liu et al., 2017b)
Polymeric NPs	1,2-distearoyl-sn-glycero-3-phosphoethanolamine-N-[methoxy (polyethylene glycol)-2000]; TPP conjugated 1,2-distearoyl-sn-glycero-3-phosphoethanolamine-N-[amino(polyethylene glycol)-2000]	Cerium(III) acetate	Neuroblastoma SH-SY5Y cell line; human glioblastoma astrocytoma (U-373); immortalized mouse hippocampal cell line(HT22); 5XFAD transgenic AD mouse model	Oxidative stress	TPP conjugated ceria NPs were reported to be biocompatible with various evaluated cell lines and also exhibit potential mitochondrial ROS scavenging activity, mitigate the reactive gliosis and suppress neuronal death – *in vitro* and *in vivo.* (Kwon et al., 2016)
Solid Lipid NPs (SLNs)	Lipids and chitosan	RVG-9R/BACE1 siRNA	Human epithelial adenocarcinoma (Caco-2) cells	Amyloid cascades	SLNs formulation improvises the charge and muco-adhesiveness of the system and relatively enhanced its drug permeability. However, further confirmation through *in vivo* tests is highly essential. (Rassu et al., 2017)
Solid Lipid NPs (SLNs)	Cetylpalmitate and functionalized with monoclonal antibody (OX26 mAb)	Resveratrol/grape seed extract	Human brain-like endothelial cells	BBB transcytosis and Amyloid cascades	Both the drug actively suppressed fibrillar formation, among them extract shows higher activity. SLNs functionalized with OX-26 antibody shown higher transcytosis compare to unfunctionalized SLNs. (Loureiro et al., 2017)
Solid lipid NPs (SLNs)	Glyceryl behenate lipids	Galantamine hydrobromide	Isoproterenol induced cognitive deficits in rats	Cholinergic systems	The SLNs loaded with galantamine, significantly restored the memory impairment in cognitive deficit rats, compare to its free form. (Misra et al., 2016)
Solid lipid NPs (SLNs)	Heparin-conjugated stearic acid; stearylamine-cationic lipid; esterquat 1	Nerve growth factor (NGF)	Induced pluripotent mouse stem cells (iPSCs)	Neuronal cell death	NGF-loaded SLNs with EQ 1 induced differentiation of neuron-like cells, which projects that these SLNs can be further adapted for neuronal regeneration studies, with potential *in vivo* evidence. (Kuo and Rajesh, 2017)
Solid lipid NPs (SLNs)	Cetyl palmitate	Rapamycin (Rp)	SH-SY5Y neuroblastoma cell line	Mammalian target of rapamycin (mTOR) signaling pathway	Rp-SLNs effectively inhibited mTOR complex 1, with a sustained release profile in neuroblastoma cells, compare to its free form. However, further *in vivo* investigation is highly essential. (Polchi et al., 2016)

## 4. Potential strategies and AD nanotherapeutics

### 4.1. Across blood brain barrier (BBB)

**Figure 2.**
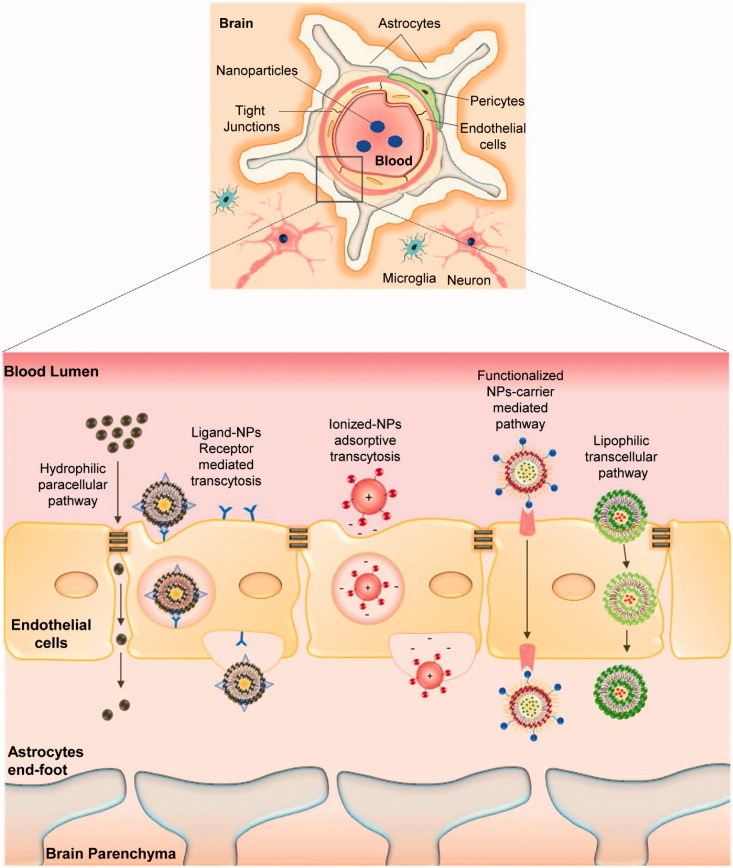
Schematic representation of potential pathways involved in nanoparticle-mediated drug trafficking across blood brain barrier associated with AD – nanotherapeutics.

CNS is a complex edifice that is bounded with extracerebral blood flow, and it is well-distinguished by specialized barriers, i.e. the blood cerebrospinal fluid barrier (B-CSF), blood brain barrier (BBB), etc. BBB plays a pivotal role in shuttling biomolecules in and out of the brain neuronal system (Pathan et al., 2009). Therefore, understanding the structural and functional characteristics of the BBB is essential to investigate and improvise drug delivery systems to the brain. BBB is mostly comprised of a vascular endothelial cell layer that is closely bound by tight junctions (TJs) and other cohesive factors (Ballabh et al., 2004). The endothelial cells are surrounded by a basement membrane covered by astrocytes end-feet and continuously monitored by surveying microglial cells (Abbott et al., 2010). Though endothelial cells are strongly bounded by numerous cohesive domains, the perseverance on selective transport of small biomolecules and cellular bodies does exist across the BBB (Ballabh et al., 2004; Abbott et al., 2010). The course of shuttling molecules through endothelial cells can be ascribed as transcytosis where highly lipophilic substances can easily pass through passive diffusion, and certain brain nutrients (i.e. glucose, amino acids) that are highly hydrophobic require the aid of special transporter proteins to pass through via active diffusion while certain larger molecules (i.e. insulin, iron transferrin) tend to pass through receptor mediated transportation (Jain, 2012). In addition, the TJs itself consists of few protein complexes, namely, occludin, claudins, and junctional adhesion molecules (JAMs), which have been reported to be likely involved in the barrier function of BBB (Wolburg & Lippoldt, 2002; Abbott et al., 2010). To surpass these barrier systems, several nano-therapeutic strategies have been adapted in the field of NP drug delivery systems (Figure 2):

- the affinity and binding of lipophilic NPs towards endothelial cells enhances the shuttling of drug candidate’s through endocytosis or lipophilic transcellular pathways;
- the adsorptive property of NPs towards blood capillaries provides a sustained release of drug candidates in the blood stream with a higher chance for drug transport across BBB;
- In addition, functionalized nanocarriers trigger receptor-mediated transcytosis and carrier protein mediated transport of drug candidates across BBB (Pathan et al., 2009; Saraiva et al., 2016).

The small-sized vesicular liposome has been extensively studied, and the FDA approved, even for commercialized use, the nanocarrier due to its self-assembling and amphiphilic properties involving safe trafficking of drug candidates (Micheli et al., 2012). Subsequently, liposomes can be readily functionalized and surface modulated using several polyether, functional proteins and cell penetrating peptides (CPPs) that aid in target-specific drug transport across BBB (Micheli et al., 2012; Rocha, 2013). For instance, Polyethylene glycol (PEG) coated liposomes were reported to successfully evade the opsonization of reticuloendothelial system (RES). In addition, glutathione-PEGylated liposomes were also reported to enhance the cellular uptake of the drug across endothelial BBB efficiently (Wong et al., 2012; Rip et al., 2014). Wang et al. successfully delivered coenzyme Q to the brain tissues of APP/PS1 double-transgenic mice and effectively suppressed the cognitive impairment using chitosan-conjugated polylactide-polyglycoside (PLGA) NPs via adsorption-mediated endocytosis across the BBB (Wang et al., 2010). Another research group successfully engineered nerve growth factor (NGF) loaded poly (butyl cyanoacrylate) (PBCA) liposomes fabricated with polysorbate 80 and demonstrated its enhanced drug delivery potential across BBB targeting cholinergic system in an amnesic rodent model (Kurakhmaeva et al., 2009).

A subsequent strategy involves binding the drug molecules with moieties such as small proteins, peptides, antibodies and trigger receptor-mediated endocytosis. CPPs such as SynB peptides, Angiopeps, etc., are widely used to transport small molecule drugs across the BBB (Orthmann et al., 2011). Sharma et al. successfully demonstrated the drug-shuttling potential of CPP modified liposomes across the brain endothelial barriers both *in vitro* and *in vivo* (Sharma et al., 2014). Mourtas et al. successfully formulated anti-transferrin tagged multifunctional liposomes and demonstrated its potential drug delivery of loaded curcumin derivatives across BBB via transferrin receptor mediated endocytosis more effectively compared to its free form. Cationic liposomes were also used to improve the permeability of loaded polyanionic DNA, RNA entities, due to its electrostatic interface with the negatively charged cell membranes and its ability to cross BBB via adsorption-mediated transcytosis or endocytosis (Joshi et al., 2014, 2015). However, certain studies reported the potentially toxic effects of cationic amine group dendrimers causing severe damage to the lipid enriched cell membranes (Thomas et al., 2009; Vidal & Guzman, 2015). Recently, a research group successfully engineered poly-amidoamine (PAMAM) dendrimers by effectively replacing surface amine groups with biocompatible hydroxyl (–OH) groups. They also demonstrated the minimal invasive nature, effective migration, and delivery of drug candidates across the BBB when administered though carotid injections in mice models (Srinageshwar et al., 2017).

On the other hand, several inorganic carrier NPs were also evaluated for their potential to cross the BBB. In general, pristine carbon nanotubes (CNTs) exhibit less biological interaction due their hydrophobic nature, and subsequent coating of the CNTs with chemical conjugates or hydrophilic biological molecules enhances the stability and functional strategies of the NPs (Bianco et al., 2005). Recently, a research group established multi-walled carbon nanotubes functionalized with amine group (MWCNTs-NH_3_+) to effectively pass through the BBB via transcytosis in both *in vitro* and *in vivo* studies (Kafa et al., 2015). Zhang et al. fruitfully conjugated wheat germ agglutinin horse radish peroxidase (WGA-HRP) to gold nanoparticles (AuNPs) and effectually exhibited the shuttling potential of the drug across the BBB through intramuscular injection in the diaphragm of rats (Zhang et al., 2016).

Following the transport of loaded drugs across the BBB, the next challenge for NPs involves targeting the drugs to the specific site of action. Few nanocarriers effectually transport the drug to specific targeted sites due to its nature, i.e. phospholipid-complexes target inflammatory sites and the reticuloendothelial system by itself (Khan et al., 2013). Some other functionalized NPs tend to adsorb an arbitrary biological entity and form a protein sheath, referred to as a ‘protein corona’, which leads to non-targeted interaction of drugs and also random deposits/accumulation of the carrier substance in biological systems (Salvati et al., 2013; Zanganeh et al., 2016). These limitations can be address either by coating the NPs with a neutral zwitterion candidate or modifying the coronated NPs to target the specific site (Zanganeh et al., 2016).

### 4.2. Amyloid cascade targets

Among the various strategies that were discussed, the interruption of Aβ fibrillar formation and Aβ plaque clearance are the most predominant therapeutic strategies to treat amyloid cascades mediated by AD pathogenesis. Several lipid-based NPs were effectively adapted in this approach due to a high binding affinity towards Aβ_1–42_ peptide. A research group functionalized liposome with phosphatidic acid and cardiolipin (Aβ_1–42_ ligands) phospholipids, effectively targeted Aβ with the highest affinity (Gobbi et al., 2010). Earlier, a homeostatic balance of Aβ peptides levels was speculated to exist between the brain and its contiguous blood circulation. By driving the brain Aβ peptides towards blood circulation, this proposed ‘sink effect’ was considered as an effective strategy to reduce the Aβ levels in the brain^139^. Recently, Ordóñez-Gutiérrez et al. (2015) decreased the Aβ levels in the brain of APP/PS1 transgenic mice through a repeated intraperitoneal injection of small liposomal vesicles functionalized with phosphatidic acid/cardiolipin, potentially altering the homeostasis of Aβ peptides in brain and circulating blood pool. Researchers also successfully engineered bifunctional liposomes, fabricated with peptide derived from apolipoprotein-E receptor-binding domain to pass across the BBB and phosphatidic acid to target Aβ, which weakens the brain Aβ aggregates and promotes Aβ clearance in APP/PS1 transgenic mice (Balducci & Mancini, 2014). Curcumin was earlier reported to effectively inhibit Aβ aggregation and enhance fibrillar (Aβ_40_) disaggregation both *in vitro* and *in vivo* (Re et al., 2010). Cheng et al. (2013). developed a highly stable nanocurcumin using a polyethylene glycol-polylactic acid co-block polymer and polyvinylpyrrolidone, which effectively transported the curcumin across BBB with around a six-fold increase in the mean residence time in the brain compared to free curcumin. Subsequently, nanocurcumin bettered the cue memory in Tg2576 mice in contextual fear conditioning tests compared to its free form Also, melatonin (a pineal gland hormone), was reported to effectively inhibit Aβ fibrillar formation through its potential interaction with Aβ_1-40_ and Aβ_1-42_ moieties (Pappolla et al., 1998). Recently, a research group developed melatonin-loaded chitosan nano-formulations that effectively exhibited anti-cancer potential in human brain tumor cell lines due to an improved cell uptake compared to its free form (Sanjeev Kumar et al., 2017). Thus, further research on effectively loading, fabricating and targeting these therapeutic small molecules towards the Aβ site, and enhancing their potential to reduce the level of amyloid burden in AD models, would be significant. Recent works adapting a hybrid methodology of loading two or more therapeutic candidates in a single NP entity provide synergy and efficacy compared to single compounds (Rodriguez-Franco et al., 2006; Gerenu et al., 2015).

**Figure 3.**
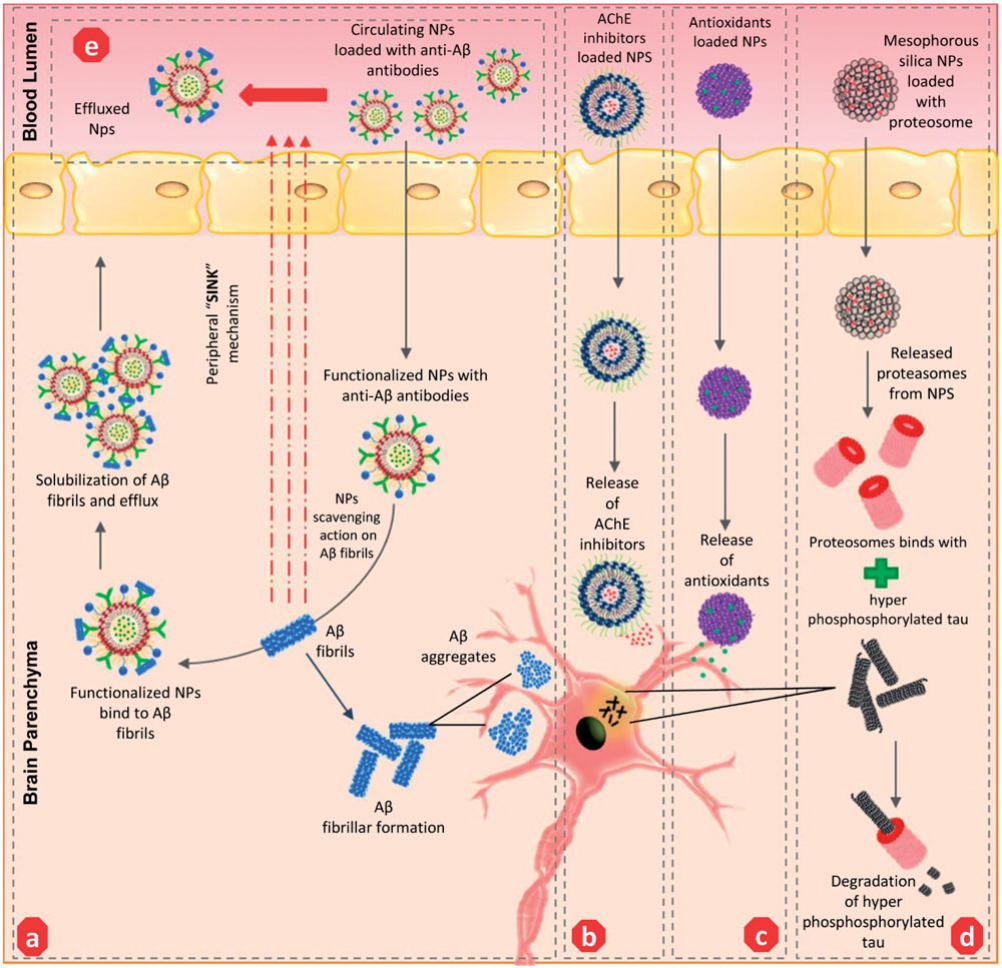
Potential mechanism of action adapted by various nanoparticles-mediated drug delivery to the targeted site of action associated with AD – nanotherapeutics: (a) anti-Aβ – functionalized NPs involves in solubilization and clearance of Aβ fibrils/aggregates, (b) AChE inhibitors loaded NPs targeting cholinergic system impairment, (c) Antioxidants loaded NPs targeting oxidative stress milieu, (d) Proteasomes loaded NPs targeting hyper-phosphorylated tau proteins, (e) anti-Aβ loaded circulating NPs initiate ‘sink mechanism’ – by captivating the Aβ fibrils from the brain to the effluence blood circulation. AChE: Acetylcholinesterase; Aβ-: amyloid beta fragment.

Another strategy involved monoclonal antibodies (MAbs) targeting Aβ fibril formation (Figure 3(a)) and sink effects (Figure 3(e)) through NP functionalization. Poduslo et al. (2011) successfully engineered monolayered phospholipid NPs fabricated with MAbs and effectually targeted against Aβ_42_ moiety in AD transgenic mice (Tg2576) model. Subsequently, other researchers re-engineered MAbs-IgG domains and fused these with transferrin ligands, which improved the drug delivery to targeted Aβ sites with an enhanced potential (Pardridge, 2015). Apolipoprotein E (ApoE), an apo class lipoprotein, and its associated polymorphic alleles were reported as a primal genetic factor in the incidental transmission of AD progression. The ApoE protein imitates lipoprotein bodies and can be easily trancytosed by endothelial cells of BBB (Liu et al., 2013). Based on this, Song et al. engineered an apolipoprotein E3-reconstituted high-density lipoprotein (ApoE3-rHDL) NP, established its potential by binding with Aβ oligomers, and facilitated its degradation by triggering glial cells. In addition, excessive non-trancytosed NPs were effectually involved in Aβ clearance in the peripheral blood pool (Song et al., 2014). Besides, inorganic carrier based gold, carbon, and silica NPs were also substantially adapted to target Aβ cascades in AD. Gao et al. (2015) recently developed a multifunctional gold NP fabricated with LPFFD peptide (β-sheet breaker peptide) and polyoxometalates-Wells Dawson structure (POMD), which revealed synergistic effects in Aβ-aggregate inhibition, Aβ fibrils dissociation and Aβ-mediated peroxidase reduction. Another research group established negatively charged carboxyl-conjugated gold nanoparticles supressed and destabilized Aβ fibrils into nebulous entities (Liao et al., 2012). In addition, carbon nanotubes (CNTs) were also adapted as effectual therapeutics against amyloid fibril formation due to its unique architecture and physical traits that enable the CN to pass through BBB and promote binding towards Aβ. Several researchers developed functionalized single-walled (SWCNTs)/multi-walled (MWCNTs) NPs and established their therapeutic efficacy against amyloidosis (Stefansson et al., 2012; Bardi et al., 2013). For instance, Xie et al. (2014) recently confirmed the inhibiting potential of SWCNTs against β-sheet formation by inducing structural shifts of Aβ_16–22_ oligomers. Excess iron (biogenic magnetite) particles were apparently found to co-exist with Aβ plaque in the brains of the AD model (Teller et al., 2015). With this ray of knowledge, a research group employed the magnetic properties of biogenic magnetite and pursued clearance of biogenic magnetite-Aβ complex (BM-Aβ) from AD brains. The team fabricated magnetic nanoparticles coated with SiO_2_ (MNP-SiO_2_) targeting the BM-Aβ complex and successfully altered the Aβ40 aggregation by peripheral exposure of extremely low-frequency magnetic field (Jia et al., 2015). However, the bio-distribution of these metallic-nanoparticles was potentially reported to cause oxidative stress, inflammation and apoptotic neuronal cell death. To overcome this issue, co-loading the metallic NPs with naturally derived antioxidant candidates or co-stimulation of endogenous antioxidant levels in the brain can possibly prevent this from happening.

### 4.3. Cholinergic impairment targets

As discussed earlier, the inhibition of AChE activity in the impaired cholinergic system of the AD model is an established therapeutic strategy against AD progression. Although rivastigmine is a well-established FDA approved AChE inhibitor, its half-life period in blood circulation is only 1.5 h, thereby reducing its efficacy (Burns, 2003; Farlow et al., 2008). Mutlu et al. encapsulated the hydrophilic rivastigmine in a sodium-taurocholate liposomal carrier and established its enhanced AChE inhibitory activity compare to its free form due to its sustained pharmacokinetic (PK) profile in an AD mouse model (Mutlu et al., 2011). Other research groups intercalated rivastigmine in a conventional cholesterol/soy-lecithinated liposomal NP formulation and demonstrated its potential in improving the half-life of the drug in the brain after intranasal administration of these NPs in an AD rat model (Arumugam et al., 2008). Jogani et al. developed tacrine-loaded propylene glycol-based formulations and its intranasal administration, effectually improving the bioavailability with reduced non-targeted tissue distribution of the drug. Galantamine, another AChE inhibitor, was successfully incorporated with l-α-phosphatidylcholine didecanoyl (DDPC)-entity based formulation, and its intranasal delivery effectively enhanced the efficacy of the drug and relatively subsided the side effects of drug (Jogani et al., 2007; Leonard et al., 2007). A few other studies have also reported to successfully intercalate naturally-derived polyphenols, which are reputed AChE inhibitors, such as epigallocatechin-3-gallate (EGCG) and catechin in PLGA, PEG, gold NPs and enhanced the bioavailability and neuroprotective efficacy compared to their free form by surpassing the physiological barriers encountered by an oral dosage regimen (Singh et al., 2016). On other hand, an exogenous supply of ACh itself to the targeted site of action radically improves the cholinergic system. However, the lower half-life of ACh and its inability to pass across BBB requires a suitable carrier system (Yang et al., 2010). Researchers have successfully developed SWCNTs loaded with ACh and have improved its safe and sustained delivery towards lysosomes as a targeted organelle, thereby suppressing the cholinergic impairment in a kainic-acid-induced the AD mice model (Yang et al., 2010). These studies proved that several nano-formulations of AChE inhibitors/ACh administered preferentially through intra-nasal delivery successfully improve the PK profile of the drug with enhanced bioavailability/efficacy and targeted, specific delivery compared to its free form (Figure 3(b)).

### 4.4. Targeting other strategies associated with AD progression

Tau aggregation is a commonly reported clinical hallmark of AD pathology. Several approaches involved in loading various tau-inhibitors in NPs have been studied. Among them, a few approaches are discussed below. Methylene blue (MB), a phenithiazone, is a well-known redox regulator and tau aggregation suppressor. However, its hydrophilicity reduces its bio-distribution in the brain, and this was effectively evaded by loading MB in hydrophobic–glutathione coated PLGA-b-PEG NPs to further exhibit a sustained release and improve the reduction in tau protein levels in *in vitro* compared to its pristine form (Jinwal et al., 2014). Early microglial activation was reported to be strongly associated with synaptic loss mediated tau tangle formation in AD animal models (Kitazawa et al., 2005; Gorlovoy et al., 2009). Based on this, Glat et al. (2013) engineered bioactive fibrin γ^377–395^ peptide intercalated in iron oxide NPs and effectively facilitated the inhibition of activated microglial cell-mediated tau tangle formation in transgenic mice. Subsequently several proteomic approaches mediating tau therapies were also investigated. In general, misfolding of functional proteins is primarily regulated by ubiquitin proteasome pathway (UPP) and molecular chaperones (Ciechanover & Kwon, 2015). In recent years, a strong linkage between UPP (26S proteasome) facilitated protein degradation, molecular chaperone upregulation and tau aggregates oligomerization was keenly recognized in AD pathogenesis (Rochet, 2007; Gadhave et al., 2016). Impairment in 26S proteasome significantly promotes tau oligomerization (Gadhave et al., 2016). Han et al. (2014) investigated and established that an exogenous supply of purified proteasome loaded in mesoporous silica NPs safely shuttles them across the BBB through endocytosis, effectually retaining its proteolytic activity and thereby suppressing the level of tau aggregates compared to the endogenous proteasome substrates (Figure 3(d)). Further study on co-loading of chaperone (heat shock proteins – HSP 70, 90) inhibitors and other tau-aggregation inhibitors in functionalized NPs can provide a way for tauopathies in AD (Blair et al., 2013).

Despite knowledge of such targets, the exact etiological factors responsible for AD have not been clearly understood. However, several other co-factorial events, i.e. oxidative stress, inflammatory response, mitochondrial dysfunction, and metal/ion disparity, were observed in the development of AD pathogenesis (Bouayed et al., 2009; Praticò, 2008). Reactive oxygen species (ROS) and reactive nitrogen species (RNS) are two commonly-documented free radical species in biological systems that have been reportedly scavenged by the endogenous antioxidant system. During physiological complications, a homeostatic imbalance occurs among the free radical formation and antioxidants, and this can lead to severe irreversible brain damage and can further escalate the progression of various neurodegenerative disorders including AD (Christen, 2000). Accumulating evidence suggests that the supply of exogenous antioxidants, such as natural flavonoids, trace elements etc., can be an effectual strategy to treat oxidative stress (Figure 3(c)) (Feng & Wang, 2012; Rahal et al., 2014). Selenium is a biological trace element that has been reported to have extensive antioxidant properties. Recently, Yin et al. (2015) successfully synthetized sialic acid modified selenium-NPs fabricated with B6 peptide, which revealed an enhanced cellular uptake and mediated bioavailability in rat adrenal medulla cells and cerebral endothelial cells, resulting in disaggregating Aβ fibrils to non-toxic oligomers more effectively than its free from. Another research group adapted Ceria (CeO_2_) NPs, well known for ROS scavenging properties, and functionalized these with triphenylphosphonium, which enables its effective localization into mitochondria and suppresses neuronal cell death in 5XFAD transgenic mice (Kwon et al., 2016). In addition, metal ions, i.e. iron, copper, zinc, and manganese, are trace elements found in the brain, which involves various neuronal functions that precisely involves in signal transduction. The loss of a homeostatic balance among these metal ions results in binding with Aβ fibrils (Maynard et al., 2005). This leads to the precipitation on Aβ fibrils and triggers fibrillar formation, oxidative stress, severe neuronal dysfunction mediated AD progression (Maynard et al., 2005; Moreira et al., 2008). To overcome this issue, chelating the metal ions has been used as an effective therapy to curb the metal-ion mediated progression of AD. Recently, Liu et al. (2017a) developed a Zn^2+^ ion specific nano-chelator where iminodiacetic acid conjugated NPs exhibit higher metal scavenging activity associated with potential inhibition of Aβ_42_ fibrillation in neuroblastoma cells. Subsequently, another research group further fabricated iminodiacetic acid-modified human serum albumin (I-HSA) conjugated NPs and established multi-faceted activities by effectively scavenging Zn^2+^/Cu^2+^-Aβ_42_ aggregates and further remodeling them to amorphous aggregates with reduced neurotoxicity (Xie et al., 2017). Zhang et al. (2017) recently developed glutaraldehyde-crosslinked chitosan NPs garnished with lysine/glutamic acid/sodium borohydride reduction, which significantly suppressed the copper oxide NPs-toxicity and effectively chelated copper ions in various cell lines. Interestingly, the binding affinity of amyloid fibril and metal ion has been utilized as transport for metal ion NPs into living cells. Bolisetty et al. (2014) utilized β-lactoglobulin (a food-protein derived amyloid fibril) in the synthesis of metal (gold, silver, and palladium) NPs that effectively helps in the internalization of these NPs into living dendritic cells.

## 5. Challenges and future perspectives

Advances in nanotechnology lead to a rise in potential therapeutic strategies against AD progression. The appropriate design and fabrication of NPs effectively surpasses conventional neurotherapeutic hurdles, i.e. oral/gastric barriers, BBB and enhances the physicochemical properties of drug candidates in biological systems. However, certain challenges persist in the field of AD nano-therapeutics. For instance, NPs itself play a dual role at the site of the Aβ fibrillar event, and a higher pool of small size NPs acts as a monomer to trigger fibrillation, whereas a pool of large surface NPs effectively adsorb free fibrils and inhibits fibrillation (Wu et al., 2008). Alternatively, other studies have reported that NPs with a smaller size and negative charge effectively shuttle across the BBB and exert more inhibitory effects (Mahmoudi et al., 2013). A few other polymeric NPs were documented to exhibit enhanced targeting and efficacy, but only with specific pH and temperature (Barua & Mitragotri, 2014). In another case, certain metallic nano-carriers were reported to facilitate Aβ fibrillation mediated AD progression and were also involved in bio-accumulation mediated neurotoxicity (Wu et al., 2008; Feng et al., 2015). These reports cumulatively suggest that several features are involved in the interplay of NPs in biological systems. Thus, an appropriate biocompatible nano-carrier fabricated with suitable size, shape and charge, and surface properties corresponding to the targeted site/mechanism of action is essential for AD nanotherapeutics. In addition, AD is a multi-faceted clinical complication, so fabricating a single NP entity with several drug molecules or multi-potential drug candidates can possibly curb the AD progression more effectively, perhaps due to synergistic effects.

Numerous *in vitro* studies have documented the potential efficacy of NPs, but limited *in vivo* investigations have been conducted. Hence, future *in vivo* investigations of these NPs can potentially reveal a long-term systemic efficacy or potential toxicity in biological systems and can be correlated with *in vitro* systems. In addition, this will also aid in obtaining the pharmaco-kinetic, pharmaco-dynamic profile of the released drugs in the biological system to opt a precise route of administration and dosage concentrations for further clinical studies. Thus, in the near future, an evaluation of the safety and efficacy of suitable NPs through human clinical trials can lead to promising, cost-effective AD therapeutics.

## 6. Conclusion

AD is one of the most commonly reported ageing related neuro-degenerative disorders, and it is characteristically diagnosed by cognitive and behavioral impairment. This clinical complication subsequently affects the routine and economic participation of an individual within a society of aging population. Though few AD therapeutics have been approved by the FDA, they were used to treat indicative problems of AD, and therapeutics to curb or stop AD progression are still under development. Here, we briefly discussed current updates on promising applications of a few nano-drug delivery systems in treatment of AD, with potential therapeutic strategies and challenges in biological systems. This can provide systematic knowledge to develop optimal NPs, targeting specific AD therapeutic strategies and paving the way to better explore the interplay of NP’s in a biological interface through future clinical/translational research.
